# Functional Heterogeneity and Context-Dependent Roles of LncRNAs in Breast Cancer

**DOI:** 10.3390/cancers17193191

**Published:** 2025-09-30

**Authors:** Shu Hui Lye, Nunaya Polycarp, Titilayomi Juliet Durojaye, Trygve O. Tollefsbol

**Affiliations:** 1 Department of Biology, University of Alabama at Birmingham, Birmingham, AL 35294, USA; slye@uab.edu (S.H.L.); npolycar@uab.edu (N.P.); jtduroja@uab.edu (T.J.D.); 2 Integrative Center for Aging Research, University of Alabama at Birmingham, Birmingham, AL 35294, USA; 3 O’Neal Comprehensive Cancer Center, University of Alabama at Birmingham, Birmingham, AL 35294, USA; 4 Nutrition Obesity Research Center, University of Alabama at Birmingham, Birmingham, AL 35294, USA; 5 Comprehensive Diabetes Center, University of Alabama at Birmingham, Birmingham, AL 35294, USA

**Keywords:** lncRNA, breast cancer, heterogeneity

## Abstract

**Simple Summary:**

This review explores the sources of functional diversity/ambiguity and context-dependent roles of long non-coding RNAs (lncRNAs) in breast cancer. LncRNAs can either promote or slow the progression of breast cancer, influenced by factors such as the tumor subtype, various cellular and molecular mechanisms, and the breast cancer tumor microenvironment. Although lncRNAs hold promise as biomarkers and therapeutic targets, their diverse nature poses challenges for clinical translation. Strategies to address this heterogeneity in lncRNAs include enhancing classification systems, utilizing technology like CRISPR/Cas tools, advanced sequencing methods, and machine learning to analyze their behavior, and—in the context of therapeutic development—focusing on strong targets.

**Abstract:**

As with other non-coding RNAs (ncRNAs), the aberrant expression of long non-coding RNAs (lncRNAs) can be associated with different forms of cancers, including breast cancer (BC). Various lncRNAs may either promote or suppress cell proliferation, metastasis, and other related cancer signaling pathways by interacting with other cellular machinery, thus affecting the expression of BC-related genes. However, lncRNAs are characterized by features that are unlike protein-coding genes, which pose unique challenges when it comes to their study and utility. They are highly diverse and may display contradictory functions depending on factors like the BC subtype, isoform diversity, epigenetic regulation, subcellular localization, interactions with various molecular partners, and the tumor microenvironment (TME), which contributes to the intratumoral heterogeneity and phenotypic plasticity. While lncRNAs have potential clinical utility, their functional heterogeneity coupled with a current paucity of knowledge of their functions present challenges for clinical translation. Strategies to address this heterogeneity include improving classification systems, employing CRISPR/Cas tools for functional studies, utilizing single-cell and spatial sequencing technologies, and prioritizing robust targets for therapeutic development. A comprehensive understanding of the lncRNA functional heterogeneity and context-dependent behavior is crucial for advancing BC research and precision medicine. This review discusses the sources of lncRNA heterogeneity, their implications in BC biology, and approaches to resolve knowledge gaps in order to harness lncRNAs for clinical applications.

## 1. Introduction

Despite progress in screening and treatment options, breast cancer (BC) continues to be among the most common cancers overall, with incidences and mortality rates projected to continue increasing globally. An analysis of estimated global cancer burden data from the GLOBOCAN database reported 2.3 million new BC cases in 2022, with annual incidence rates rising by 1–5% in approximately half of the 185 countries assessed [1]. Like with many cancers, BC is characterized by complex etiologies, consisting of multiple subtypes with various clinical presentations. As such, a comprehensive understanding of this disease is essential for improving early detection strategies and developing effective therapeutic and preventive interventions. An area of interest that may shed light on the regulatory complexities underlying mammary oncogenesis is the study of long non-coding RNAs (lncRNAs), a distinct subset of non-coding RNAs (ncRNAs).

### 1.1. LncRNAs: A Brief Overview

LncRNAs are commonly defined to be non-coding transcripts of over 200 nucleotides (nt). Due to the existence of other well-characterized ncRNAs of sizes that approach this 200 nt border, recommendations have also recently been made to redefine lncRNAs to include transcripts largely generated by RNA polymerase II that are over 500 nt [2]. The vastly heterogenous nature of lncRNAs is exemplified by efforts by multiple groups at subcategorizing lncRNAs. In addition to length, for instance, lncRNAs may be classified according to their various molecular characteristics, including the sequence elements, function, subcellular localization, association with other components of cellular components, and biological contexts [3]. Recognizing that the function of an lncRNA can vary depending on the tissue, disease stage, or molecular context is pertinent when it comes to characterizing their roles in cancer biology and for identifying which lncRNAs may serve as reliable biomarkers or therapeutic targets.

#### 1.1.1. LncRNAs: Biogenesis

Like messenger RNAs (mRNAs), lncRNAs are often transcribed by RNA polymerase II, 5′ capped (at 7-methylguanosine) and 3′ polyadenylated, and can be alternatively spliced [2]. LncRNA transcription often occurs at nucleosome-depleted regions, which facilitates chromatin accessibility and R-loop formation [4]. According to the GENCODE consortium’s annotation of human lncRNA, the vast majority of lncRNA introns are flanked by canonical splice sites (GT/AG) [5]. However, many lncRNAs lack polyA tails. They are spliced more inefficiently than mRNA due to a weak internal splicing signal and significant distances between their 5′ splice site, 3′ splice site, and junction (branch point) [6,7]. lncRNA transcripts are expressed at relatively low levels and tend to exhibit more limited temporal restriction compared to mRNA [2]. Their exon architecture may differ from that of protein-coding genes, with 42% of lncRNA transcripts containing only two exons, compared to just 6% of protein-coding gene transcripts [5]. Short sequences within lncRNAs may predict where they localize and how they interact with proteins, unlike mRNAs which are largely present in the cytoplasm [8].

#### 1.1.2. LncRNAs: Functions

Compared to other forms of ncRNAs like microRNAs, which are primarily involved in gene silencing an active RNA-induced silencing complex (RISC), lncRNAs appear to be subject to a greater degree of functional ambiguity. By interacting with components of cellular machinery, including but not limited to proteins, other RNA, and DNA, lncRNAs may be involved in the regulation of the cellular function at multiple regulatory levels. This includes their ability to act as dynamic scaffolds, decoys, and guides that engage with diverse molecular partners, including other DNA, RNAs, and proteins [9,10,11,12,13]. For instance, lncRNAs can guide chromatin-modifying complexes to specific genomic loci to epigenetically silence or activate transcription [14,15,16,17]. They function as decoys by sequestering transcription factors or microRNAs (miRNA), thereby preventing them from binding to their native targets [18,19]. Furthermore, their role as scaffolds enables them to bring together multiple proteins to form functional ribonucleoprotein complexes that influence processes such as mRNA splicing, stability, and translation [20]. Through these fundamental mechanisms, scaffolding, decoying, and guiding lncRNAs integrate signals and modulate critical cellular pathways in BC, including proliferation, apoptosis, and metastasis [13,21].

Consequently, efforts have been made to investigate the role of lncRNAs in cancer pathogenesis. Computational analyses have identified lncRNAs to be significantly implicated in cancer regulation. For example, some long intergenic non-coding RNAs (lincRNAs) may act as universal cancer repressors [22]. In BC patients, the aberrant expression of lncRNAs has been reported [23]. The molecular actions of lncRNAs in BC may lead to either the suppression or facilitation of BC cell proliferation, metastasis, the epithelial–mesenchymal transition (EMT), and even resistance to drugs used in the treatment and management of BC, like tamoxifen and adriamycin [24].

Despite the reported roles of lncRNAs as versatile regulators of gene expression, many other studies show contradictory behavior and a lack of fixed functionality. As an example, homeotic transformation and skeletal malformation was reported in mice with the homozygous deletion of the lncRNA HOX Transcript Antisense RNA (*HOTAIR*) [25]. However, a later study using the same mutant mice crossed to a different genetic background failed to recapitulate the effects of the *HOTAIR* knockout [26]. Another widely studied lncRNA, X-inactive specific transcript (*XIST*), is identified as an oncogene that is often overexpressed in many cancers, but may act as a tumor suppressor in some cases [27,28].

### 1.2. Breast Cancer: Pathophysiology

The etiology of BC is an ongoing area of study. Most BCs manifest as invasive carcinomas, while a smaller but still significant percentage are lobular carcinomas [29]. Only about 10% or fewer BC cases are due to heritable genetic mutations, the majority of which are found in the tumor suppressor genes *BRCA1* and *BRCA2* [29]. Over 90% of BCs are considered sporadic. Explanations for pathogenesis or risk factors include hormonal imbalances, immune system influences, and lifestyle factors [30,31,32]. Of significance is the role of estrogen in BC, which promotes cell proliferation in mammary epithelial tissue. Estrogen can also lead to oncogenesis by promoting chromosomal translocations and subsequent copy number amplification events, particularly in genomic regions where estrogen receptors (ERs) bind [33]. Additionally, BC cells interact dynamically with the tumor microenvironment (TME), which consists of immune cells, the extracellular matrix (ECM), hypoxic regions, and other players. Exposure to compounds due to the environment or lifestyle, like certain nutrients or carcinogens, for instance, could change the TME by regulating lymphocyte activity or creating hypoxic conditions, affecting BC outcomes [30,34].

In conjunction with the complex molecular dynamics underlying the BC etiology, the heterogeneity of the disease itself is highlighted by its classification into various subtypes. BCs can be classified by their histological grade, Ki-67 proliferative index, and the expression of three receptors: the estrogen receptor (ER), human epidermal growth factor receptor 2 (HER2), and progesterone receptor (PgR). Further molecular classification groups BCs into Luminal A, Luminal B, HER2-enriched, Basal-like, and Normal-like. Luminal A tumors typically show smaller sizes and lower grades and are characterized by favorable long-term patient survival among BC subtypes [35]. Compared to Luminal A, Luminal B tumors have increased proliferative properties and present worse overall survival. HER2-enriched tumors also often present with higher grades and aggressive features, while TNBC presents with genomic instability along with high aggressiveness and limited therapeutic options [35].

LncRNAs may be involved in BC pathophysiology by regulating signaling pathways such as the MAPK cascade and PI3K/PTEN/AKT/mTOR pathway [36]. Large-scale analyses demonstrate the association of lncRNA expression with specific breast cancer subtypes [32]. For example, ER-positive tumors are enriched for lncRNAs linked to estrogen signaling, while ER-negative and Basal-like tumors show lncRNA signatures related to immune activation and NF-κB signaling. Additionally, lncRNAs expressed in fibroblasts contribute to ECM remodeling, particularly in Luminal A tumors, indicating the influence of the TME.

## 2. Materials and Methods

The sourcing of relevant research and references in this work was carried out via the appraisal of multiple primary empirical articles and review articles. The searches for these articles were primarily carried out using the PubMed database, presently maintained by the United States National Library of Medicine (NLM). Google Search and Google Scholar are also web engines employed for the searches. Keywords utilized in the creation of this work largely include but are not limited to “breast cancer” and “lncRNA”. Clinicaltrials.gov was also utilized, wherein “breast cancer” was the term entered for the “Condition/disease” filter and “lncRNA” for the “Intervention/treatment” filter. No descriptive statistical analyses nor meta-analyses were conducted in the creation of this work.

## 3. Sources of LncRNA Functional Heterogeneity

### 3.1. Breast Cancer Subtypes

LncRNA expression patterns exhibit greater subtype specificity than protein-coding genes (PCGs), with 18.27% of lncRNAs showing subtype-specific expression compared to 10.55% of PCGs [37]. Certain lncRNAs appear to have distinct expression patterns in specific BC subtypes: *TSIX* is unique to Luminal A, *PVT1* to Luminal B, *SNHG8* and *GAS5* to HER2-enriched tumors, and *HCP5*, *SNHG3*, and *MIR155HG* to Basal-like or triple-negative breast cancer (TNBC) subtypes (Table 1) [38,39,40]. These subtype-specific lncRNAs influence critical cancer-related pathways, such as immune modulation, cell cycle progression, and EMT, underscoring their potential in precision medicine. Some lncRNAs, such as *HOTAIR*, *EMX2OS*, and *MYCNOS*, are expressed across all subtypes, indicating a more general role in BC biology or involvement in cellular processes that are common to diverse tumor types (Table 1) [38].

Among research groups who identified multiple lncRNAs specific to certain BC subtypes is Wasson et al., who analyzed the expression of over 12,000 lncRNAs across BC subtypes using TCGA and TANRIC data (Table 1). The authors revealed subtype-specific associations with patient survival and that select lncRNAs promote proliferation in subtype-matched BC cells. Of note, poorer patient survival linked to *LINC01269* expression was observed exclusively in the HER2-positive (HER2+) BC subtype [41]. Consistent with this association, reduced cell proliferation was only observed upon *LINC01269* knockdown in HER2+ BC cell lines. Targeting *LINC01269* together with HER2 antagonists like trastuzumab produces additive effects on reducing cancer cell viability. Also identified by the authors was the lncRNA *AL078604.2*, which is associated with poor prognoses, specifically in TNBC patients, and only had a negative effect on TNBC cell proliferation upon knockdown [41]. Interestingly, this particular investigation reported that lncRNAs commonly studied in BC like *HOTAIR*, *NEAT1*, and *MALAT1* were not significantly linked to patient survival outcomes [41].

Hyaluronan synthase 2 antisense RNA 1 (*HAS2-AS1*) is an lncRNA with low expression in ER+ tumors and high expression in ER- tumors. However, this high expression is correlated with the better survivability of patients with ER- BC, whereas the same is not observed in patients with ER+ BC (Table 1) [42]. Two-dimensional cell culture experiments appear to recapitulate this finding, whereby *HAS2-AS1* overexpression decreases the cell viability and invasiveness of ER- cells, but not ER+ cells [42]. Thus, *HAS2-AS1* may function as a tumor suppressor specifically in ER- BC. It could also function as a miRNA sponge and behave as an epigenetic regulator [43].

*LINC00324*, *PTPRG-AS1*, and *SNHG17* are three examples of lncRNAs significantly associated with both ER+ and ER- subtypes and the tumor’s histologic grade (Table 1). Their expression profiles enable risk stratification for relapse-free survival. Specifically, high *LINC00324* and low *PTPRG-AS1* and *SNHG17* expression correlate with favorable clinical outcomes [44]. Of particular interest, *PTPRG-AS1* is an antisense lncRNA to PTPRG, a known tumor suppressor in multiple cancers. The upregulation of *PTPRG-AS1* may lead to the suppression of *PTPRG*, potentially contributing to tumor progression [44]. The unsupervised clustering of lncRNA expression aligns closely with PAM50 molecular subtypes, underscoring their utility in refined molecular subtyping [44,45]. For instance, basal-like/triple-negative tumors exhibit distinct lncRNA expression profiles from other subtypes and normal tissue, indicating the potential of lncRNAs for precise subtype classification and personalized treatment.

Another example of an lncRNA relevant to BC is *MNX1-AS1*, which is significantly upregulated in TNBC tissues and cell lines, correlating with poor survival outcomes (Table 1) [46]. Mechanistically, *MNX1-AS1* promotes tumor growth and metastasis by enhancing the phosphorylation of the transcription factor Stat3, thereby activating the JAK/Stat3 signaling pathway crucial for TNBC progression. Silencing *MNX1-AS1* in vitro and in vivo reduces tumor proliferation and invasion, underscoring its potential as a TNBC-specific therapeutic target [46].

Further emphasizing the therapeutic relevance of lncRNAs in TNBC, a clinically guided siRNA screening approach identified *BCAL8* (breast cancer-associated lncRNA 8) as a functional driver of tumor proliferation (Table 1). *BCAL8* may promote cell-cycle progression by upregulating Cyclin E2. In MDA-MB-231 cells, the knockdown of *BCAL8* led to reduced cell proliferation in vitro and reduced tumor growth in xenograft models via the inhibition of the G1–S cell-cycle transition [37]. Elevated *BCAL8* expression and genomic gain are also associated with poor prognosis in BC.

**Table 1:** Representative lncRNAs linked to overall and subtype-specific breast cancers, with corresponding expression patterns and prognostic correlations.

BC Subtype(s)	Associated LncRNA	Expression in BC Samples or Cells	BC Prognosis	References
All BCs	*BCAL8*	Upregulated	Linked to poor prognosis	[37]
	*EMX2OS*	Downregulated	Unknown correlation	[38]
	*MYCNOS*	Upregulated	Unknown correlation	[38]
	*NEAT1*	Frequently overexpressed; some mixed results	Frequently linked to poor prognosis; some mixed results	[41,47,48,49,50]
	*MALAT1*	Frequently upregulated; some mixed results	Frequently linked to poor prognosis; some mixed results	[41,49,50]
	*HOTAIR*	Frequently upregulated; some mixed results	Frequently linked to poor prognosis; some mixed results	[41,50,51,52]
Luminal A	*TSIX*	Downregulated	Unknown correlation	[38]
Luminal B	*PVT1*	Upregulated	Unknown correlation	[38]
HER2-enriched	*SNHG8*	Downregulated	Unknown correlation	[38]
	*GAS5*	Downregulated	Linked to resistance to trastuzumab and lapatinib	[38,44]
	*LINC01269*	Upregulated	Linked to poor prognosis	[41]
ER-	*HAS2-AS1*	Upregulated	Linked to better prognosis	[42]
ER+	*HAS2-AS1*	Downregulated	Weak correlation	[42]
ER+ and ER-	*LINC00324*	Upregulated	Linked to better prognosis	[44]
	*PTPRG-AS1*	Downregulated	Linked to better prognosis	[44]
	*SNHG17*	Downregulated	Linked to better prognosis	[44]
Basal-like/TNBC	*HCP5*	Upregulated	Linked to better prognosis	[38,39]
	*SNHG3*	Upregulated	Linked to poor prognosis	[38,40]
	*MIR155H*	Upregulated	Unknown correlation	[38]
	*AL078604*	Upregulated	Linked to poor prognosis	[41]
	*MNX1-AS1*	Upregulated	Linked to poor prognosis	[46]

### 3.2. Isoform Diversity and Alternative Splicing

Alternative splicing (AS) can contribute to the functional heterogeneity of lncRNAs in BC by generating multiple transcript variants (isoforms) with different regulatory capacities, localization patterns, or interaction partners. While lncRNAs are spliced similarly to mRNAs, they are much less efficiently spliced, which may lead to a greater number of spliced lncRNA transcripts per exon [6]. However, increasing the thymine content in the polypyrimidine tract (PPT) of the 3′ splice site has been experimentally shown to enhance lncRNA splicing by creating a stronger binding site for splicing factors, thus facilitating increased lncRNA splicing efficiency [53]. Should splicing hold greater functional significance for lncRNAs over evolutionary time, their splicing efficiency and subsequent function could potentially become altered via PPT mutations, biasing the production of certain lncRNA isoforms. Currently, it is known that lncRNAs transcripts and their variants are often produced by Alternative Last Exons in both humans and mouse models [6].

**Figure 1:**
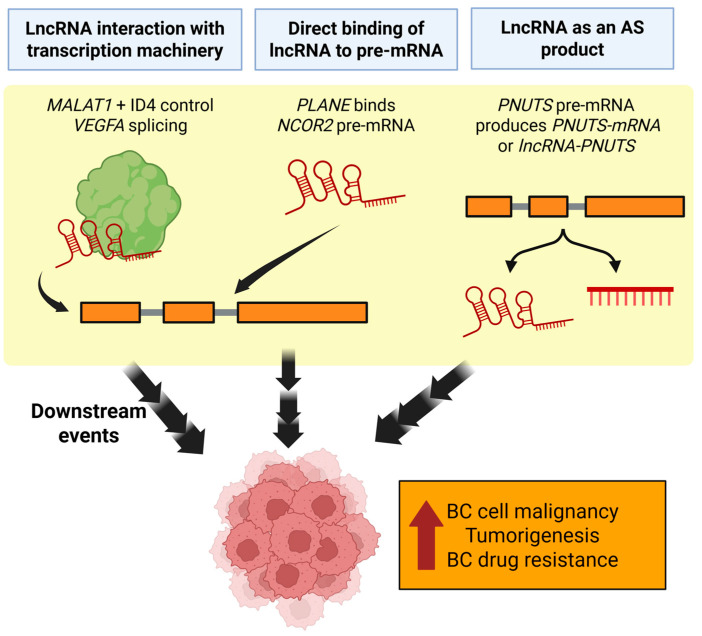
Example mechanisms of breast cancer malignancy regulation by lncRNAs via control of alternative splicing. *MALAT1* and *PLANE* may affect alternative splicing by indirect and direct binding of other breast-cancer related genes, respectively. When *PNUTS* alternative splicing is biased towards its lncRNA form, tumorigenesis is promoted. AS = Alternative splicing; BC = Breast cancer. Created in https://BioRender.com (Accessed on 19 August 2025).

LncRNAs can themselves influence the AS of other genes (Figure 1). *MALAT1*, for example, interacts with several different splicing factors and transcriptional regulators. Turco et al. demonstrate that *MALAT1* and ID4 work together to control *VEGFA* splicing, particularly favoring isoforms that lack exon 7 [54]. This interaction additionally results in the back-splicing of the *VEGFA* exon 7, generating a circular RNA, *circ_0076611* which promotes TNBC cell malignancy by facilitating the translation of select mRNAs [54]. Another example is the lncRNA *ZNF649-AS1*, which is found to promote trastuzumab resistance in HER2-positive BC [55]. *ZNF649-AS1* regulates the AS of *EXOC7*, likely by facilitating its interaction with pre-mRNA processing factor 8 (PRPF8). This leads to PD-L1-mediated immune evasion, establishing a tumor microenvironment (TME) that compromises the trastuzumab effectiveness [55].

*LncRNA-PNUTS* is an AS product of the *PNUTS* pre-mRNA, which can give rise to *PNUTS-mRNA* or *lncRNA-PNUTS*. The heterogeneous nuclear ribonucleoprotein E1 (hnRNP E1) controls this splicing decision, whereby its absence shifts splicing towards the production of *lncRNA-PNUTS*. *miR-205* preferentially binds to the lncRNA form over the mRNA form of *PNUTS*, which correlates with the upregulation of ZEB proteins and increased tumorigenesis in mesenchymal breast tumor cells [56].

Some lncRNAs also affect AS by binding to target pre-mRNAs. One such example is *PLANE*, which is the longest isoform of the lncRNA *MELTF-AS1*, and was found to be highly expressed in multiple cancer cell lines. *PLANE* directly binds to and forms an RNA-RNA duplex with the *NCOR2* pre-mRNA at intron 45, cooperating with hnRNPM and thereby directing the AS of *NCOR2* to generate lncRNAs that promote tumorigenicity [57].

In addition to AS, some studies have reported differential behavior or an abundance of lncRNA isoforms in the context of BC.

#### 3.2.1. HOX Antisense Intergenic RNA (*HOTAIR*)

Among the most studied lncRNAs is *HOTAIR*, which is positioned in the *HOXC* locus on human chromosome 12q13, implicating its role in the regulation of body patterning and development. *HOTAIR* interacts with proteins, RNA, and DNA, forming scaffolds that enable the formation of gene regulatory complexes [51]. High *HOTAIR* expression is prevalent in many human cancers [50,51,58]. In BC, its expression is associated with decreasing levels of tumor suppressors p53 and p21, and the induction of the *c-Myc* oncogenic pathway. In addition, *HOTAIR* can act as competing endogenous RNA (ceRNA), sequestering *miR-331-3p* which normally suppresses HER2 expression [51].

*HOTAIR* is commonly studied in the context of its canonical isoform (*HOTAIR-C*), which is a 6-exon 2364 nt transcript. Two other isoforms, *HOTAIR-N* and *HOTAIR-U*, have also been reported [59,60]. Data from TCGA suggest that *HOTAIR-N* is the predominant isoform upregulated in breast carcinomas and is associated with poor overall survival [60,61]. Structurally, the *HOTAIR-N* promoter consists of a strong CpG island containing G-quadruplex (G4) motifs [60]. This CpG island of the *HOTAIR-N* promoter in BC cells appears to contribute to *HOTAIR-N* expression control via DNA and histone modifications [60]. Also notable is the fact that unlike observations in patient breast tumor samples, 2D BC cultures exhibit low *HOTAIR* expression. In Claudin-low TNBC cells cultured in a laminin-rich extracellular matrix (lrECM), *HOTAIR-N*, but not *HOTAIR-C*, is robustly upregulated due to the epigenetic activation of its promoter, mediated by canonical ECM signaling pathways [61]. This induction of *HOTAIR-N* is required for invasive growth in the 3D culture, implicating its role in translating ECM signals into metastasis.

#### 3.2.2. Nuclear Paraspeckle Assembly Transcript 1 (*NEAT1*)

A highly abundant lncRNA often upregulated in many cancers including BC, *NEAT1*, localizes with paraspeckles, which are subnuclear bodies involved in gene expression via the sequestration of mRNAs [47,48,50,62,63]. The *NEAT1* lncRNA is transcribed from the *NEAT1* gene on chromosome 11q13.1, and encodes two major isoforms: the short isoform, *NEAT1_1* (3.7 kb), and *NEAT1_2* (23 kb) [64]. While *NEAT1_1* is involved in gene regulation, *NEAT1_2* is reported to be involved in the structural integrity of paraspeckles, subnuclear membraneless organelles [65]. In mice, *Neat1_1* is more highly expressed in most tissues and better conserved. Upon the loss of *Neat1_1* via the deletion of its polyadenylation signal, *Neat1_1* undergoes forced switching to *Neat1_2* [66].

Knutsen et al. reported RNA-FISH analyses carried out on formalin-fixed paraffin-embedded (FFPE) needle biopsies using probes specifically targeting *NEAT1_2*. From there, it was found that *NEAT1_2* was more highly expressed in HER2-positive (HER2+) and luminal B subtypes of breast cancers [67]. Interestingly, *NEAT1_2* expression is significantly lower in ER+ tumors compared to ER- tumors, with the inverse being true for *NEAT1_1* [67].

It has been demonstrated that knocking out *NEAT1_1*, but not *NEAT1_2*, had no observable effect on the proliferation of osteosarcoma and colon cancer cells. Additionally, *Neat1_1*-specific knockout mice do not exhibit the defects observed in *Neat1* KO mice, implying *NEAT1_1* as nonessential in the context of cancer cell growth regulation [49]. However, a later study showed that when *NEAT1_1* was knocked down in the ER+ BC MCF7 cells, the cells exhibited decreased proliferation [68]. In MMTV-PyVT mice, *Neat1_1* inactivation significantly delays mammary tumor onset [68].

#### 3.2.3. Metastasis-Associated Lung Adenocarcinoma Transcript 1 (*MALAT1*)

The *MALAT1* lncRNA, which is also known as *NEAT2*, is transcribed from the *MALAT1* gene located in the 11q13 locus. This lncRNA is derived from the tRNA processing of the *MALAT1* primary transcript, in which another ncRNA, mascRNA, is formed. Patients with TNBC exhibit high levels of *MALAT1* and a poor prognosis [50]. Mechanistically, *MALAT1* may promote metastasis by inducing the epithelial–mesenchymal transition (EMT) via PI3K-Akt pathway activation [69].

Meseure et al. identified two *MALAT1* isoforms from the dbEST database, namely the full-length transcript (*MALAT1-FL*) and an alternatively spliced variant (*Δsv-MALAT1*). Unlike *MALAT1-FL*, *Δsv-MALAT1* expression varied in the BC tumor samples [70]. Patients with tumors expressing low levels of the *Δsv-MALAT1* isoform also showed reduced metastasis-free survival (MFS), indicating this particular isoform’s potential as a prognostic marker [70]. Meseure et al., therefore, proposed that *Δsv-MALAT1* may function as a molecular decoy, sequestering interaction partners normally bound to *FL-MALAT1*, which would disrupt its regulatory activity [70].

### 3.3. Epigenetic Regulation as a Driver of Functional Heterogeneity

**Figure 2:**
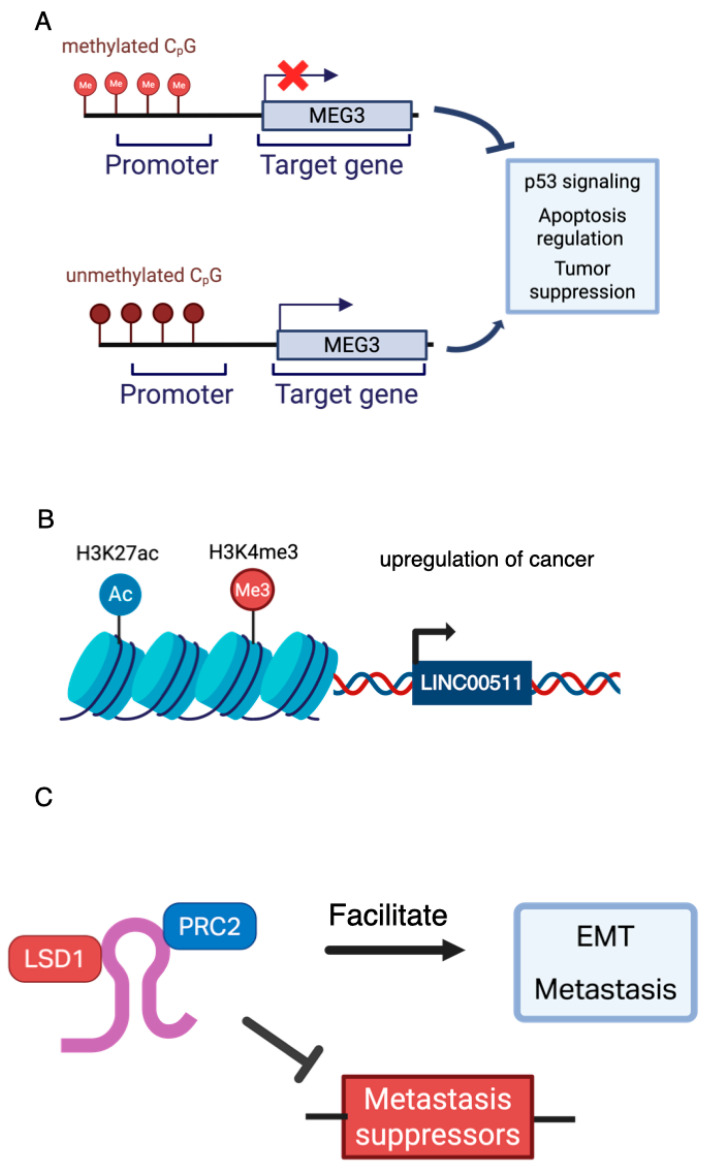
Epigenetic regulation mechanisms of lncRNAs in breast cancer. (**A**) The lncRNA *MEG3* is frequently silenced in BC due to promoter methylation. When unmethylated, *MEG3* can contribute to p53 signaling. (**B**) The *LINC00511* genomic superenhancer regions are enriched with H3K27ac and H3K4me3, which can lead to upregulation of stem-like cancer cells. (**C**) *HOTAIR* recruits PRC2 and LSD1, reprogramming chromatin to silence metastasis suppressor genes and promoting oncogenesis. EMT = Epithelial–mesenchymal transition. Created in https://BioRender.com (Accessed on 19 August 2025).

Epigenetic modifications, which are inheritable yet reversible changes in gene expression that do not involve alterations in the DNA sequence, play a crucial role in regulating the activity of lncRNAs in cancer [71]. In BC, DNA methylation, histone modifications, and chromatin remodeling profoundly influence the transcriptional landscape and context-specific functionality of lncRNAs, contributing to intratumoral heterogeneity and phenotypic plasticity [72,73]. Promoter methylation is one of the most studied mechanisms. The tumor suppressor lncRNA *MEG3*, for example, is frequently silenced in BC due to promoter hypermethylation, which disrupts its role in p53 signaling and apoptosis regulation [74]. Likewise, the reversal of this methylation has been shown to inhibit EMT and suppress tumor progression (Figure 2A) [54].

Histone modifications also regulate lncRNA expression. Superenhancer-associated lncRNAs such as *LINC00511* are enriched with H3K27ac and H3K4me3 marks, supporting their upregulation in stem-like cancer cells and linking them to a poor prognosis (Figure 2B) [75]. Chromatin looping further shapes lncRNA behavior by altering isoform expression and promoter–enhancer contacts across BC subtypes.

Importantly, lncRNAs not only respond to epigenetic cues, but actively shape them. For example, *HOTAIR* functions as a scaffold, recruiting the epigenetic regulators PRC2 and LSD1 to reprogram chromatin and repress metastasis suppressor genes, facilitating EMT and metastasis (Figure 2C) [76]. LncRNAs function as ceRNAs by absorbing miRNAs that regulate EMT-TFs, thereby enhancing the plasticity in response to environmental stress [12].

The dynamic and context-specific roles of lncRNAs emphasize the importance of epigenetic regulation in determining their function. Grasping these interactions is essential for creating subtype-specific biomarkers and developing lncRNA-targeted treatments in precision oncology.

### 3.4. Subcellular Localization

Unlike mRNAs, which are transported from the nucleus to the cytoplasm for translation, lncRNAs may be largely localized in the nucleus or be shuttled into the cytoplasm. LncRNAs may also be transcribed in the mitochondria by mitochondrial genomes or associated with other organelles including ribosomes, the endoplasmic reticulum, and endosomes [77]. Some of the shuttling of lncRNAs between compartments may be dependent on the cellular context.

The majority of lncRNAs can predominantly be found in the nucleus, where they regulate gene expression through chromatin remodeling, transcriptional control, and RNA processing. An example includes the long Intergenic Non-Protein Coding RNA 839 (*LINC00839*), which is localized in the nucleus in BC cell lines [78]. The upregulation of *LINC00839* is associated with poor prognosis in BC patients in addition to chemoresistance [78]. In vitro evidence indicates that overexpressing *LINC00839* enriches c-Myc protein levels via a feedback loop, whereby Myc reinforces *LINC00839* upregulation by binding to the *LINC00839* promoter region. Downstream events modulate PI3K/AKT signaling, contributing to dysregulated BC cell oncogenecity [78]. As Myc is primarily localized in the nucleus, the nuclear localization of *LINC00839* is consistent with its potential involvement in oncogenesis.

Other lncRNAs may not remain localized to one area. *H19*, which is transcribed from an imprinted locus on chromosome 11p15, can be found in the nucleus, but has a primarily cytoplasmic localization. The high expression of *H19* correlates with increased breast tumorigenesis [79]. Mechanistically, *H19* can act as an endogenous sponge, which is a feature of many other cytoplasmic lncRNAs. In particular, *H19* sequesters microRNAs from the let-7 family, reducing the let-7 availability, which subsequently increases LIN28 [79]. LIN28 itself inhibits the maturation of let-7, creating a double-negative feedback loop with let-7 and a double-positive feedback loop with *H19*. This regulatory circuitry promotes breast cancer stem cell (BCSC) maintenance [79].

### 3.5. Interactions with Different Molecular Partners

The functional versatility of long non-coding RNAs (lncRNAs) in BC arises largely from their capacity to engage with diverse molecular partners, including microRNAs (miRNAs), proteins, and other RNA or DNA molecules. These interactions allow lncRNAs to modulate the cellular behavior across multiple regulatory levels, such as transcription, splicing, translation, and chromatin remodeling [12,13].

#### 3.5.1. LncRNA–miRNA Interactions

LncRNAs can function as ceRNAs, which is often described as “miRNA sponging” [80]. ceRNAs can sequester miRNAs through shared miRNA response elements (MREs), preventing these miRNAs from binding to and repressing their target mRNAs [80]. This interaction effectively derepresses miRNA targets, leading to the upregulation of specific genes that can drive oncogenic pathways. The oncogenic lncRNA *LINC00511* exemplifies this principle: the direct binding of *LINC00511* to tumor-suppressive miR-185-3p liberates the transcription factor E2F1 from miR-185-3p-mediated repression, elevating E2F1 protein levels. This transcriptionally activates key cell cycle regulators, including cyclin-dependent kinase genes CCNA2 and CCNE1, which promote uncontrolled cellular proliferation in BC [80].

The case of the lncRNA *PVT1* also highlights the contextual complexity of ceRNA networks: while *PVT1* functions as a ceRNA for the miR-200 family, which is critical for maintaining an epithelial phenotype in normal breast tissue, this regulatory interaction is frequently disrupted in breast tumors despite high *PVT1* expression [81]. This functional decoupling suggests that the efficacy of a ceRNA is not solely determined by its abundance, but is also influenced by the cellular environment. Potential explanations include post-translational modifications of the core RISC components (e.g., Ago2) that alter the binding affinity [82] or saturation of the ceRNA network by other RNA transcripts with higher affinity for the shared miRNA, effectively outcompeting *PVT1* in the tumor context [81].

#### 3.5.2. LncRNA–Protein Interactions

Beyond miRNA interactions, lncRNAs serve as guides and scaffolds for proteins, directing their activity to specific genomic loci or facilitating the assembly of macromolecular complexes [83]. This capacity to act as modular platforms is central to their roles in epigenetic remodeling and the organization of nuclear architecture, processes frequently dysregulated in cancer. *HOTAIR*, for example, functions as a modular scaffold that simultaneously interacts with distinct histone-modifying complexes via independent domains [17]. Specifically, its 5′ domain binds to the Polycomb Repressive Complex 2 (PRC2), which catalyzes the trimethylation of histone H3 on lysine 27 (H3K27me3) [16]. Concurrently, its 3′ domain recruits the LSD1/CoREST/REST complex, which demethylates histone H3 on lysine 4 (H3K4me2). By co-targeting these two complexes to the promoters of metastasis suppressor genes, such as those within the HOXD cluster, *HOTAIR* orchestrates a bivalent epigenetic switch that stably silences transcription. This mechanism is a key driver of BC invasion and metastasis, correlating with poor patient prognosis.

As previously discussed, the functional diversity of lncRNAs is exemplified by *NEAT1*, which exists in two distinct isoforms with divergent roles. *NEAT1_2* acts as a critical architectural scaffold, recruiting and organizing essential RNA-binding proteins such as NONO and SFPQ to form the core of these nuclear bodies. Under cellular stress conditions, including hypoxia, a common feature of tumors, paraspeckles rapidly enlarge and sequester various nucleic acids and proteins, including transcription factors and hyperedited mRNAs [84]. This sequestration acts as a post-transcriptional regulatory mechanism, modulating the transcriptome to promote cell survival and suppress apoptosis in BC.

#### 3.5.3. Multiplex and Dynamic Behavior

Many lncRNAs function within multiplex networks, simultaneously interacting with proteins, miRNAs, and chromatin [85,86]. The lncRNA *XIST* provides an example of multiplexing. While its primary role is to silence one X chromosome in females, its mechanism involves coordinated action across multiple domains. *XIST* recruits repressive complexes like PRC2 to deposit H3K27me3 across the chromosome, while simultaneously interacting with proteins involved in nuclear compartmentalization, orchestrating structural and epigenetic change [87]. A more direct example relevant to cancer is the lncRNA *H19*, which has been shown to function as a ceRNA for miRNAs like *let-7*, while its spliced product can also bind to and modulate the stability of proteins such as p53, demonstrating how a single locus can yield molecules with multiplex functions [88]. Post-transcriptional modifications such as N6-methyladenosine (m6A) methylation can also alter the lncRNA stability, localization, and interaction partners. For example, the m6A modification of the lncRNA *LINC01133* has been shown to affect its ability to sponge miRNAs and promote cancer metastasis, adding a dynamic layer of regulation [89,90]. Interpreting these intricate and dynamic interactomes necessitates the application of advanced methodologies.

### 3.6. Hypoxic Tumor Microenvironments

The BC tumor microenvironment (TME) is the dynamic ecosystem that surrounds and interacts with breast tumor cells. In addition to the malignant epithelial cancer cells, it comprises non-cancerous cell types, including fibroblasts, immune cells, and endothelial cells, as well as various signaling molecules and the extracellular matrix (ECM). Hypoxia is a classic microenvironmental feature that shapes solid tumor progression and heterogeneity. Hypoxia can arise from several factors, including exposure to carcinogens, the tumor cell-driven remodeling of the TME, and elevated metabolic activity in cancer cells. These factors may increase the distance between tumor cells and blood vessels, reducing oxygen diffusion [34]. In response to hypoxic stress, cancer cells can change their behavior, complicating the effectiveness of treatments like chemotherapy [34]. LncRNAs may play an intermediary role between the TME and BC cells by being regulated by hypoxia or by regulating hypoxia signaling [91].

**Figure 3:**
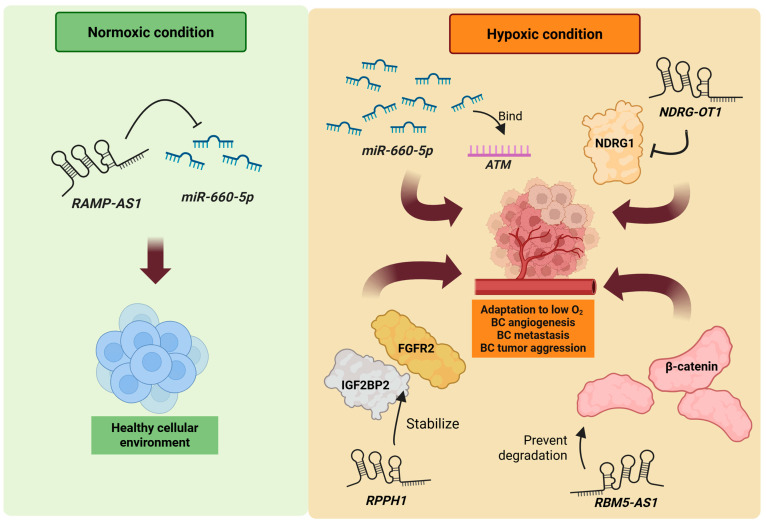
Hypoxic conditions influence lncRNA expression and subsequent downstream events related to BC etiology. *RAMP-AS1* expression inhibits *miR-660-5p* from interacting with *ATM* mRNA, maintaining normal cellular environments. *NDRG-OT1*, *RBM5-AS1*, and *RPPH1* are examples of lncRNAs that are upregulated in hypoxic conditions. These lncRNAs are involved in mechanisms including stabilization of oncogenic factors or inhibition of certain targets, which promote BC pathogenesis. Created in https://BioRender.com (Accessed on 19 August 2025).

*RAMP2-AS1* was identified as a hypoxia-suppressed lncRNA in BC through in silico analyses and in vitro experiments. Unlike many lncRNAs, its upregulation in BC patients is associated with favorable prognostic outcomes. Mechanistically, hypoxia reduces *RAMP2-AS1* levels, potentially via a *RAMP2-AS1*/*miR-660-5p*/*ATM* axis in which fewer *RAMP2-AS1* molecules are available to sequester *miR-660-5p*, freeing the latter to bind to the *ATM* mRNA (Figure 3) [92]. Similarly, N-Myc Downstream Regulated Gene 1-Overlapping 1 (*NDRG1-OT1*) was identified through the expression profiling of MCF7 BC cells exposed to varying oxygen levels. Its dysregulation under hypoxia suggests a role in the cellular adaptation to low-oxygen stress (Figure 3) [93]. In contrast, *RBM5-AS1* is consistently induced under hypoxic conditions and is significantly upregulated in both BC cells and tumor tissues, implying a more active role in tumor adaptation and possible aggressiveness (Figure 3) [94]. Another hypoxia-responsive lncRNA, *RPPH1*—a component of the RNase P complex—is also upregulated in hypoxic environments (Figure 3) [95]. Its expression is associated with poor prognosis in BC patients, suggesting that distinct hypoxia-regulated lncRNAs may exert opposing effects on tumor progression depending on their molecular targets and mechanisms of action.

### 3.7. Hormone Signaling

ER and PgR signaling are the primary pathways underpinning hormone-responsive BCs. One component of the TME can be cancer-associated fibroblasts (CAFs), which can remodel the ECM and secrete paracrine factors that can alter estrogen receptor signaling. This influences intratumoral heterogeneity by supporting distinct cancer cell subpopulations with variable hormone dependence [96]. The lncRNA *HOTAIR* is overexpressed in ER+ BCs and may interact with estrogen signaling pathways by promoting chromatin remodeling and the transcriptional activation of estrogen-responsive genes [97]. Similarly, *LINC00472* expression is associated with favorable outcomes in ER+ BC and appears to be regulated by estrogen levels [98]. By modulating these hormone-driven pathways, lncRNAs help maintain heterogeneous BC cell populations with varied hormone sensitivities, facilitating the adaptation of these cells to fluctuating microenvironmental conditions.

## 4. LncRNAs in the Clinic: Biomarker and Therapeutic Development in Breast Cancer

LncRNAs present a compelling avenue for the development of disease-related biomarkers and targeted intervention strategies. Unlike tissue biopsies, which are invasive and can pose significant discomfort and risks to patients, the extraction of lncRNAs from biofluids—such as blood, plasma, or serum—offers a minimally invasive alternative for cancer diagnostics. Tumor cells can release endogenous lncRNAs into human bodily fluids as extracellular vesicles (EVs), protecting the lncRNAs from degradation [52].

LncRNAs have also gained attention as potential therapeutic targets, especially with the rapid progress in antisense oligonucleotide (ASO) technologies. ASOs and small interfering RNAs (siRNAs) that mediate RNA interference (RNAi) are examples of these approaches, which aim to specifically modulate or silence lncRNAs that contribute to tumor progression, metastasis, or therapy resistance. Specifically, ASOs and siRNA molecules can be delivered to patients to selectively bind and degrade pathogenic lncRNA transcripts [99].

As several computational, in vitro, and in vivo studies have drawn promising correlations between specific lncRNA expression levels and patient prognoses, researchers began touting lncRNAs like *HOTAIR*, *H19*, and *MALAT1* for their potential as diagnostic biomarkers or therapeutic targets (Table 2) [100]. LncRNAs may also predict patient responses to drug treatments. For instance, a reduction in *PRRT3-AS1* is associated with paclitaxel resistance, while the absence of *GAS5* is linked to resistance to trastuzumab and lapatinib [101,102].

In a study published by the Zhimin Shao group, an integrated signature consisting of three mRNAs and two lncRNAs was developed using transcriptome arrays to classify TNBC patients based on the disease recurrence risk [103]. The researchers then carried out a randomized phase 3 trial, in which patients classified as “high risk” based on the integrated signature exhibited comparable survival when treated with intensive adjuvant chemotherapy compared to if they were treated with standard adjuvant chemotherapy [104]. This series of studies provides a case for the use of integrated lncRNA signatures to predict patient outcomes and tailor chemotherapy options.

Besides the efforts of some researchers, there are limited data available on the specific utility of lncRNAs in the clinic; much less so in the context of BC [100]. Indeed, the study of lncRNAs is still an area of significant evolution whereby their structure and function are not well-understood. The development of clinical applications for lncRNAs in BC is hinged on a good understanding of lncRNA mechanisms and robust data derived from functional lncRNA studies. To further evolve the field, there is a need to shift focus toward understanding the fundamental mechanisms governing the lncRNA biology in BC. Advancing clinical applications will require multidisciplinary efforts to clarify how lncRNAs contribute to genome organization, nuclear architecture, and regulatory networks.

## 5. Strategies to Address LncRNA Functional Heterogeneity

### 5.1. Improving LncRNA Classification Systems

Due to the structural and functional diversity of lncRNAs, coupled with an ongoing evolution of lncRNA research, current lncRNA classification systems will require an increasingly comprehensive framework. To date, the GENCODE system provides among the most extensive and up-to-date source for lncRNA annotations and for understanding how they are classified based on their genomic context, including their relationship to protein-coding genes (e.g., antisense, intronic, and intergenic), exon–intron structure, and transcriptional evidence [5]. The current GENCODE release (version 49) includes 35,899 lncRNAs across reference chromosomes, scaffolds, assembly patches, and alternative loci [105]. In addition to the GENCODE catalog, similar classification efforts by several other groups have also been carried out. Prominent examples include LNCipedia, NONCODE, and LncBook, which also offer detailed annotations of human lncRNAs based on multiple criteria like genomic location, multi-omic data integration, function predictions [106,107,108].

However, the continually evolving landscape of lncRNA research can cause instances of misannotation or inconsistency. GENCODE has continually updated names of transcripts, and some protein-coding genes (PCGs) have been reclassified as lncRNAs over different versions. In one example, an analysis of GENCODE version 24 reports the case of *C3orf10*, which was originally classified as a PCG, but later annotated as an lncRNA, then back to PGC before being dropped from the database [109]. This dynamicity highlights both the complexity of transcriptome characterization and the challenges in achieving stable, standardized annotations for lncRNA species as more data become available.

There is, hence, a need for the continual and active refinement of lncRNA annotation. Recently, GENCODE has focused on creating their catalog of full-length lncRNAs using Capture Long-read Sequencing (CLS) instead of short-read RNA-seq methods, enabling improved annotation quality (Table 3) [110,111]. This method involves a capture array with probes targeting known and potential lncRNA regions in human and mouse genomes. cDNA libraries were prepared and enriched for complete 5′ to 3′ RNA molecules using CapTrap-Seq. Then, long-read sequencing was carried out on the libraries [111].

Machine learning (ML) and large language models (LLM) can also serve as assets in enhancing lncRNA annotation (Table 3). These methods are suited to addressing the complex semantic nature of lncRNAs by leveraging vast amounts of sequence data to uncover patterns that traditional methods and manual annotation may overlook [117]. For example, TERIUS is an ML tool that implements a two-step filtration process that distinguishes bona fide lncRNAs from false positives, particularly addressing issues with 3′ UTR fragments [118]. An example of an LLM tool is LncRNA-BERT, which uses a model pre-trained on RNA datasets from multiple databases, allowing it to classify lncRNAs effectively without relying on predefined features [119]. Incorporating lncRNAs into multi-omic predictive models has improved molecular classification. ML techniques using lncRNA signatures have demonstrated high precision in differentiating BC subtypes, particularly in distinguishing Basal from HER2-enriched groups. Visualization methods like t-SNE support these findings, highlighting the subtype-specific clustering ability of lncRNAs [120].

### 5.2. Uncovering LncRNA Functions with CRISPR/Cas Systems

Loss-of-function and gain-of-function experiments are key tools used to elucidate the function of a gene or a protein. Similarly, such experiments can be used to study the various functions of lncRNAs. In the past decade, CRISPR/Cas9 technology has seen a boom, gaining popularity as a tool used for numerous applications ranging from in vitro functional knockout studies to CRISPR-edited CAR-T cells, which can be tailored to target and kill cancer cells. However, while CRISPR/Cas9 genome editing can delete lncRNA genes or regulatory regions, its use is limited in this case because many lncRNA loci overlap or neighbor other genes, risking unintended effects on those genes. Thus, small interfering RNA (siRNA) and antisense oligonucleotides (ASOs) methods remain widely used in the study of the lncRNA function [121]. These methods are not without their disadvantages: RNAi relies on the RNA-induced silencing complex (RISC), which primarily operates in the cytoplasm. CRISPR systems are not bound by this limitation and can operate both in the nucleus and the cytoplasm [122]. ASOs degrade RNA by forming DNA-RNA hybrids recognized by RNase H1, but they require the complex delivery of modified oligonucleotides. In addition, siRNA and ASO methods for functional studies do not give rise to a complete loss of function [122].

CRISPR/Cas9 can be used to target lncRNA genes, creating a double-stranded break (DSB) which is repaired with a single guide RNA (sgRNA) template, which can be customized to induce insertions or deletions of DNA (Table 3). As discussed, this method risks affecting adjacent genes. Hence, CRISPR interference (CRISPRi) or CRISPR activation (CRISPRa) tools can be used instead to repress or activate genes without inducing a DSB (Table 3) [122]. Instead, these tools utilize a dead Cas9 protein (dCas9) to block RNA polymerase II from binding or to recruit transcription activators to a desired region. It should be noted that the dCas9 protein construct could cause unintended alterations of transcriptional contexts by affecting nearby PGCs. While the utilization of these CRISPRi or CRISPRa still induces fewer overall large off-target effects compared to RNAi, these tools are unable to distinguish cis- from trans-acting lncRNA functions [123]. There is another CRISPR system which can directly target lncRNA molecules, which uses Cas13 as a RNA-cleaving enzyme. The CRISPR/Cas13 system enables the study of lncRNA loci that may be located close to each other or to PGCs [124]. While the use of Cas13 constrains the sgRNA design due to its preference for regions containing protospacer flanking sequences, the system has shown similar overall efficiency to RNAi methods with fewer off-target effects [122]. Notably, CRISPR/Cas13 has been used to successfully knock down very long intergenic non-coding RNAs (vlincRNAs), supporting its utility in functional phenotypic studies of lncRNAs across the whole cell (Table 3) [114].

In a study using BC cells, three CRISPR/Cas9 techniques were used to prevent the transcription of the lncRNA *LINC00511*. Subsequent functional assessments led to the assessment that *LINC00511* promotes BC cell proliferation and malignancy [112]. CRISPRa has also been employed to study the function of lncRNA OBSCN-Antisense RNA 1 (*OBSCN-AS1*) in the context of BC. When activated, *OBSCN-AS1* restores *OBSCN* expression, which suppresses cell migration in TNBC cells [113]. Another study used a CRISPR-Cas13d screen on previously discovered lncRNAs linked to BC to look for lncRNAs that upregulate cell proliferation. The authors identified the lncRNA *KILR*, which was highlighted as a tumor suppressor that limits proliferation by sequestering the DNA replication factor *RPA1* [114].

### 5.3. Use of Single-Cell and Spatial Sequencing in LncRNA Research

LncRNAs exhibit highly context-specific and cell-type-specific expression patterns, posing challenges for traditional bulk RNA-seq approaches that average out signals across heterogeneous cell populations. Studying lncRNAs in the context of solid cancers adds another layer of complexity because tumors consist of diverse malignant, stromal, and immune cell populations. By enabling the analysis of transcriptional profiles at the resolution of individual cells, single-cell RNA sequencing (scRNA-seq) has emerged as a tool to assist in the detection of heterogeneous and cell-specific lncRNA expression patterns within tumors (Table 3). For example, studies in TNBC xenografts reveal that most lncRNAs are expressed at low but detectable levels, with some lncRNAs such as *MALAT1* and *NEAT1* widely expressed, while others are restricted to distinct subpopulations [115].

Spatial transcriptomics complements scRNA-seq by preserving the spatial context of gene expression. This enables the preservation of the spatial context of lncRNA expression within BC specimens and potentially mapping lncRNAs to specific tumor niches (Table 3). The utilization of a spatial atlas can enable the identification of circulating biomarker candidates. For example, Xu et al. carried out a spatial transcriptomic analysis, identifying *HULC* lncRNA abundance in both normal and hepatocellular carcinoma tissue, but at markedly higher levels in the latter [125]. Since *HULC* is also highly detectable in both liver and blood samples, it is a potential candidate for a non-invasive biomarker for liver cancer [125]. While spatially resolved data on lncRNA targets in BC are limited, similar principles of spatial transcriptomics can be applied to inform biomarker discovery in the context of BC.

Using single-cell and spatial transcriptomics data, Pakrithi et al. identified over 35,000 novel lncRNAs in tissues across 13 types of cancers. The authors note that lncRNAs such as *MALAT1* and *NORAD* are broadly and abundantly expressed across many tissue types, indicating low tissue specificity [116]. Other lncRNAs, like *TINCR*, are highly expressed only in BC samples, indicating the importance of considering the tissue specificity in lncRNA research [116]. The annotations and visualizations of lncRNA expression profiles for each tissue were made available on SPanC-Lnc—the Spatial and Single-Cell Pan-Cancer Atlas of lncRNAs —an interactive web browser tool [116].

### 5.4. Prioritizing LncRNA Targets

A certain level of discernment is warranted when prioritizing lncRNA as potential candidates for therapeutic development. While computational analyses of publicly available datasets can be carried out that show significant correlations between lncRNAs and patient survival, observations should be replicable in model systems. As such, functional screens and studies should be conducted across multiple contexts and not limited to single cell lines [126]. To alleviate points of ambiguity present in current lncRNA studies, Ponting and Haerty recommend prioritizing targets that exhibit high sequence conservation, abundance in multiple cell types, specific subcellular localization, and observable interaction with other molecules [126].

Personalized lncRNA expression profiling methods may also be a way to identify potential lncRNA targets relevant to different patients given the variability of lncRNA expression (Table 3). Zhao et al. developed LncRNA Individualization (*LncRIndiv*), a personalized lncRNA expression profiling method which generates sample-specific lncRNA signatures known as individualized differentially expressed lncRNA (IDElncRNA) [101]. The *LncRIndiv* pipeline uses lncRNA expression profiles from TCGA to construct a IDElncRNA profile. Expression ranks of lncRNA pairs from TCGA normal samples are used as a reference standard; then, each patient’s tumor sample from TCGA is compared to this reference to detect individualized lncRNA deregulation to generate IDElncRNA profiles. This approach overcomes the limitations of population-level analyses and accounts for the data heterogeneity, normalization issues, and batch effects.

## 6. Conclusions

Many studies have demonstrated the association of specific lncRNA expression with outcomes ranging from cancer cell behavior in vitro to patient survival and responses to drug therapy. The ease of obtaining lncRNAs through minimally invasive sampling makes them attractive candidates for clinical use in BC, which include applications such as lncRNA-based diagnostics, the direct inhibition of lncRNAs, or their use as therapeutic agents to influence the cancer cell behavior. Despite these promising avenues, challenges remain in effectively translating lncRNA-based therapies into clinical practice due to an incomplete understanding of the lncRNA function and heterogeneity of lncRNA expression. The functional heterogeneity and context dependence of long noncoding RNAs (lncRNAs) is crucial for advancing cancer research. Their diverse modes of action, cell-type specificity, and dynamic responses to environmental or pathological cues can significantly influence the tumor behavior and therapeutic outcomes. Hence, a more comprehensive understanding of lncRNA heterogeneity and their context-dependent behavior is necessary in order to better address the research gaps. This is possible through evolving technologies like CRISPR systems and next-generation sequencing techniques, coupled with persistent efforts with regards to lncRNA classifications. Additionally, therapeutic development efforts in the lncRNA field should prioritize targets supported by rigorous, reproducible evidence from multiple independent studies.

## Figures and Tables

**Table 2:** Widely studied lncRNAs with known functions, prognostic relevance, and therapeutic potential.

LncRNA	Function	BC Prognosis	Therapeutic Potential	References
* HOTAIR *	Scaffolds PRC2/LSD1, drives chromatin remodeling, EMT, and metastasis	Upregulation frequently linked to poor prognosis; some mixed results	ASOs/siRNA to disrupt PRC2/LSD1 interaction; *HOTAIR-N* upregulation as a candidate biomarker	[61,76]
* NEAT1 *	Maintains paraspeckle integrity, promotes stress survival	Upregulation frequently linked to poor prognosis; some mixed results	Isoform-specific targeting; RNA-FISH for patient stratification	[63,66,67]
* MALAT1 *	Regulates EMT, metastasis, alternative splicing, PI3K-Akt signaling	Upregulation frequently linked to poor prognosis; some mixed results	*Δsv-MALAT1* as decoy; *Δsv-MALAT1* downregulation as a candidate biomarker	[49,69,70]
* H19 *	Sponges let-7; supports stemness; confers tamoxifen resistance via autophagy regulation	Upregulation frequently linked to poor prognosis	*H19* upregulation as a candidate biomarker	[79,88]
* GAS5 *	Tumor suppressor, modulates drug sensitivity (trastuzumab, lapatinib)	Downregulation linked to drug resistance in HER2+ BC	Restoration strategies to enhance HER2-targeted therapy	[38,102]

**Table 3:** List of technologies or methods that can be used to resolve knowledge gaps related to lncRNA functional heterogeneity.

Tool/Technique	Overview	Example Use Case in BC	References
Personalized lncRNA expression profiling	Generates of sample-specific lncRNA (IDElncRNAs).	*LncRIndiv* constructs a IDElncRNA profile for BRCA to characterize subtype-specfic lncRNAs.	[101]
Capture Long-Read Sequencing (CLS)	Probes targeting lncRNAs are created, from which cDNA libraries enriching for complete 5′ to 3′ RNA molecules are then made. Long-read sequencing is then carried out on the libraries.	Improve lncRNA annotation quality.	[111]
CRISPR/Cas9	DSB induced by Cas9 is repaired by sgRNA, which can be customized to make specific edits to DNA.	Suppression of *LINC00511* transcription, which decreases BC cell proliferation rate.	[112]
CRISPRa	CRISPR system to activate genes by using catalytically inactive Cas9 to recruit transcriptional activators.	Activate *OBSCN-AS1* to restore *OBSCN* expression, thus suppressing TNBC cell migration.	[113]
CRISPRi	CRISPR system to repress gene expression by using catalytically inactive Cas9 to recruit transcriptional repressors.		
CRISPR/Cas13d	Knock-down lncRNA via cleavage by Cas13.	Used in a screen for lncRNAs that can upregulate BC cell proliferation. The tumor suppressor *KILR* was identified via this method.	[114]
scRNA-seq	Analyses of transcriptional profiles at the resolution of individual cells.	Together with spatial transcriptomics, *TINCR* expression was found to be highly expressed in BC samples.	[115,116]
Spatial transcriptomics	Preserves the spatial context of gene expression within samples with heterogenous cell populations.	Together with scRNA-seq, *TINCR* expression was found to be highly expressed in BC samples.	[116]
Machine learning (ML) methods	The use of computational models and statistical algorithms to automatically analyze and draw inferences from patterns.	Uncover patterns overlooked by traditional annotation. TERIUS distinguishes lncRNAs from false positives by focusing on 3′ UTR fragments.	[117,118]
Large Language Models (LLMs)	Artificial intelligence system that automatically analyzes and generates human-like language and text based on patterns from large text datasets that it is trained on.	Uncover patterns overlooked by traditional annotation. LncRNA-BERT uses a model trained on existing sequence databases to distinguish mRNA from lncRNA.	[117,119]

## Abbreviations

AS	Alternative splicing
ASO	Antisense oligonucleotide
BC	Breast cancer
BCSC	Breast cancer stem cell
CAF	Cancer-associated fibroblast
ceRNA	Competing endogenous RNA
CLIP-seq	Crosslinking immunoprecipitation with high-throughput sequencing
CLS	Capture long-read sequencing
c-Myc	Cellular myelocytomatosis oncogene
DSB	Double-stranded break
ECM	Extracellular matrix
EMT	Epithelial–mesenchymal transition
EMT-TF	Epithelial–mesenchymal transition transcription factor
ER	Estrogen receptor
ER+	Estrogen receptor-positive
ER-	Estrogen receptor-negative
EV	Extracellular vesicle
FFPE	Formalin-fixed paraffin-embedded
HER2	Human epidermal growth factor 2
HER2+	Human epidermal growth factor 2-positive
H3K27ac	Histone H3 lysine 27 acetylation
H3K4me3	Histone H3 lysine 4 trimethylation
IDElncRNA	Individualized differentially expressed lncRNA
LLM	Large language model
lncRNA	Long non-coding ribonucleic acid
lrECM	Laminin-rich extracellular matrix
MFS	Metastasis-free survival
miRNA	Micro-ribonucleic acid
ML	Machine learning
mRNA	Messenger ribonucleic acid
nt	Nucleotide
PgR	Progesterone receptor
RBP	RNA-binding protein
RISC	RNA-induced silencing complex
RNA-FISH	RNA-fluorescence in situ hybridization
RNAi	RNA interference
scRNA-seq	Single-cell RNA sequencing
siRNA	Small interfering RNA
TANRIC	The Atlas of Noncoding RNAs in Cancer
TCGA	The Cancer Genome Atlas
TNBC	Triple-negative breast cancer
